# Vitamin C Status and Cognitive Function: A Systematic Review

**DOI:** 10.3390/nu9090960

**Published:** 2017-08-30

**Authors:** Nikolaj Travica, Karin Ried, Avni Sali, Andrew Scholey, Irene Hudson, Andrew Pipingas

**Affiliations:** 1 Centre for Human Psychopharmacology, Swinburne University of Technology, John St, Hawthorn, Melbourne 3122, Australia; andrew@scholeylab.com (A.Sc.); Ihudson@swin.edu.au (I.H.); apipingas@swin.edu.au (A.P.); 2 The National Institute of Integrative Medicine, 21 Burwood Rd, Hawthorn, Melbourne 3122, Australia; karinried@niim.com.au (K.R.); asali@niim.com.au (A.Sa.)

**Keywords:** vitamin C, ascorbic acid, central nervous system, cognition, Alzheimer’s, dementia, MMSE

## Abstract

Vitamin C plays a role in neuronal differentiation, maturation, myelin formation and modulation of the cholinergic, catecholinergic, and glutaminergic systems. This review evaluates the link between vitamin C status and cognitive performance, in both cognitively intact and impaired individuals. We searched the PUBMED, SCOPUS, SciSearch and the Cochrane Library from 1980 to January 2017, finding 50 studies, with randomised controlled trials (RCTs, *n* = 5), prospective (*n* = 24), cross-sectional (*n* = 17) and case-control (*n* = 4) studies. Of these, 36 studies were conducted in healthy participants and 14 on cognitively impaired individuals (including Alzheimer’s and dementia). Vitamin C status was measured using food frequency questionnaires or plasma vitamin C. Cognition was assessed using a variety of tests, mostly the Mini-Mental-State-Examination (MMSE). In summary, studies demonstrated higher mean vitamin C concentrations in the cognitively intact groups of participants compared to cognitively impaired groups. No correlation between vitamin C concentrations and MMSE cognitive function was apparent in the cognitively impaired individuals. The MMSE was not suitable to detect a variance in cognition in the healthy group. Analysis of the studies that used a variety of cognitive assessments in the cognitively intact was beyond the scope of this review; however, qualitative assessment revealed a potential association between plasma vitamin C concentrations and cognition. Due to a number of limitations in these studies, further research is needed, utilizing plasma vitamin C concentrations and sensitive cognitive assessments that are suitable for cognitively intact adults.

## 1. Introduction

The biological benefits of the water soluble molecule vitamin C (l-ascorbic acid or ascorbate) have been well documented [1,2,3,4,5]. Based on its unique chemistry, the biological role of ascorbate is to act as a reducing agent, donating electrons in various enzymatic and non-enzymatic reactions [6]. It is a cofactor for at least eight enzymatic reactions involved in key bodily processes including the production of collagen, preventing harmful genetic mutations, protecting white blood cells [7] and the production of carnitine, vital for energy [8]. Ascorbate is reversibly oxidized with the loss of two electrons to form dehydroascorbic acid (DHAA).

Despite the extensive research into its enzymatic roles and antioxidant properties, the biological roles of vitamin C on the brain have only recently been described in detail. Animal studies have explored this biological link. In particular, research has focused on guinea pigs, due to their inability to biosynthesize vitamin C from glucose, similar to humans [9]. As a result of this biological limitation, the human brain relies on dietary sources of vitamin C. Animal studies have shown that vitamin C plays a vital role in neurodevelopment by influencing neuronal differentiation and the general development of neurons and myelin formation [9]. Additional, specific neurotransmitter functions include modulation of the cholinergic, catecholinergic, and glutaminergic systems of the brain. Ascorbic acid affects synaptic neurotransmission by preventing neurotransmitter binding to receptors [10], by modulating their release and reuptake [11], and also acting as a cofactor in neurotransmitter synthesis [12]. Another neuromodulatory role of Vitamin C appears to be its involvement in presynaptic re-uptake of glutamate [13], exhibiting a direct effect in the prevention of neuronal over-stimulation by glutamate [14].

Less research has been conducted on ascorbate in collagen synthesis in brain than in other organs, but minimal amounts are essential for blood vessel formation (angiogenesis). Vitamin C is essential for the formation of procollagen which then acts as an intracellular “glue” that gives support, shape and bulk to blood vessels [15]. Studies indicate that vitamin C deficiency in the brain is associated with a reduction in angiogenesis and vascular dysfunction [16,17] and the production of nitric oxide, responsible for vasodilation.

Neurons are especially sensitive to ascorbate deficiency, possibly due to 10-fold higher rates of oxidative metabolism than supporting glia [18]. Ascorbate at the concentrations present in CSF and neurons in vivo has been shown to effectively scavenge superoxide [19]. Once a superoxide radical is formed in the mitochondria of neurons, ascorbate catalyses its conversion to H_2_O_2_ and is oxidised in the process to an ascorbate free radical and DHAA. Ascorbate also supports the regeneration of other antioxidants, such as vitamin E and glutathione [19].

Indicative of its vital role in the brain is its recycling, homeostatic mechanism [20] which maintains vitamin C concentrations in the brain and neuronal tissues relative to other bodily organs and tissues. In the healthy brain, the content of vitamin C in cerebrospinal fluid (CSF) is highly concentrated compared to plasma (2–4 times more, 150–400 µmol/L) [21]. In whole brain, 1 to 2 mM of ascorbic acid has been detected, while intracellular neuronal concentrations are much higher, reaching up to 10 mM [22]. These high concentrations are the result of DHAA being recycled into ascorbate within astrocytes, which consist of glutathione [23]. The most saturated vitamin C brain regions include the cerebral cortex, hippocampus and amygdala [24,25].

Although higher plasma ascorbic acid concentrations generally result in higher CSF concentrations, these concentrations start to reach a steady state. As plasma concentrations decline, relatively more ascorbate is pumped into the CSF in order to maintain homeostasis [26]. Studies have demonstrated a higher CSF: plasma ratio in those with lower plasma vitamin C [26,27]. This could be a reflection of the increased “consumption” of ascorbate by the oxidative stressed brain, leading to lower plasma concentrations [26].

Thus, not only is it difficult to deplete brain ascorbate, it is also difficult to increase levels above those set by uptake and recycling mechanisms. In neuronal cells, the apparent Michaelis–Menten transport kinetics (K_m_) for ascorbate appears to be somewhat high (113 µmol/L); this affinity corresponds well to plasma ascorbate concentrations of 30–60 μmol/L [28]. Thus, plasma vitamin C can only relate to brain vitamin C status in a narrow window, likely levels below 30 μmol/L.

Duration of deficiency has shown to influence brain ascorbate concentrations to a higher degree than the amount of depletion. This is exemplified by observations in acute scurvy where brain concentrations of ascorbate are relatively maintained through depletion of peripheral tissues [29], whereas marginal deficiency for longer periods of time resulted in greater brain ascorbate depletions [30].

Given the various biological roles on the central nervous system, a number of studies have been conducted with the intention of exploring whether vitamin C status is associated with cognitive performance in cognitively intact participants as well as those diagnosed with a neurodegenerative condition. This systematic review is the first to explore the effects of blood vitamin C status and cognitive performance in both cognitively impaired and intact groups of participants. This systematic review summarises current knowledge and provides recommendations for future studies.

## 2. Methods

### 2.1. Search Strategy

We searched the PUBMED, SCOPUS, SciSearch and the Cochrane Library for publications from 1980 to January 2017. Keywords used were vitamin C, ascorbic acid, antioxidant, cognition, memory, Alzheimer’s and dementia. Additional published reports were obtained by checking references of screened articles. Studies only examining cognitive function and vitamin C status were included.

### 2.2. Selection of Trials

Study designs included randomised controlled trials, prospective cohort, cross-sectional, and case-control, restricted to those in the English language. This selection included adult participants who were either cognitively intact or diagnosed with a neurodegenerative condition such as Alzheimer’s or dementia. Studies that administered some form of vitamin C measure and quantitative cognitive assessment were accepted.

### 2.3. Quality Assessment 

Quality of studies was independently assessed by two investigators (NT and KR). Appraisal was determined using established guidelines for randomised, controlled trials (RCT), and observational studies (prospective and cohort) established from the Cochrane collaboration [31]. Quality was assessed on selection bias, allocation bias, attrition bias, methods to control confounding factors, and conflict of interest. Compliance was further assessed in RCTs. Higher-quality trials (score ≥4 of 8 points for RCT, ≥3 of 4 points for prospective and ≥2 of 3 for cross-sectional and case control) were compared with lower-quality studies.

### 2.4. Analysis of Trials Using Comparable Methods

An initial survey of the literature revealed that many studies used comparable cognitive and vitamin C measures—The Mini Mental State Examination (MMSE) and blood plasma vitamin C concentrations. Given this consistency in measurement we decided to further explore these trends across studies. A brief summary of these inclusions and methods is presented below. We contacted authors for mean values and standard deviations of studies which did not report numerical mean vitamin C concentrations or MMSE scores (0–30) but instead placed the means into categories (e.g., MMSE score of over/under 27, vitamin C concentrations into deficient/adequate ranges).

### 2.5. Blood Plasma Vitamin C 

Given the practicality and accuracy of measuring absorbed vitamin C status through blood plasma, plasma vitamin C has been considered the ideal measure of vitamin C status [32]. A number of investigated studies have used this measure to determine vitamin C status. vitamin C blood concentrations, based on population studies, indicate that a plasma concentration of <11 μmol/L is considered to be deficient, 11–28 μmol/L is depleted or marginally deficient, 28–40 μmol/L is adequate, and >40 μmol/L is optimal [33]. Other studies measured CSF vitamin C concentrations or incorporated a variety of FFQs and supplementation questionnaires, measuring daily intake in milligrams. A recommended daily intake of 200 mg/day has been suggested, as this corresponds with optimal vitamin C blood concentrations [34].

### 2.6. Measure of Cognition 

The MMSE is a simple validated and reliable paper and pen questionnaire designed to estimate the severity and progression of cognitive impairment and used to follow the course of cognitive changes in an individual over time [35]. Any score greater than or equal to 24 points (out of 30) indicates normal cognition. Below this, scores can indicate severe (≤9 points), moderate (10–18 points) or mild (19–23 points) cognitive impairment [36]. The cognitive domains measured include attention and calculation, recall, language, ability to follow simple commands and orientation. Descriptive analyses were conducted for all included studies, which assessed vitamin C concentrations (means and standard deviations in µmol/L for blood tests and mg/day for FFQs), and mean MMSE scores.

### 2.7. Z Statistical Analysis-Correlation Between Blood Vitamin C and MMSE Score 

Using IBM SPSS (version 23, Chicago, IL, USA) *t*-tests were conducted, comparing the baseline blood vitamin C concentrations and baseline MMSE scores between cognitively intact and impaired participants. Due to the ordinal nature of MMSE scores and ratio scales for blood test concentrations, a Spearman’s correlation coefficient analysis (*r* values) was conducted. R-squared values, assessing goodness of fit and test of normality were conducted to establish the correlation between mean vitamin C concentrations and MMSE scores.

Only studies which measured blood vitamin C concentrations and cognition through the MMSE were compared. Comparable mean vitamin C blood concentrations and MMSE scores were extracted as separate data points from each of the studies and plotted graphically. A number of studies assessing cognitively impaired individuals also used healthy controls. The mean MMSE and vitamin C concentrations from these controls was added to the mean scores of other cognitively intact samples for comparison.

FFQ-based vitamin C levels were also converted to predicted blood concentrations, where every 1.97 mg of consumed vitamin C equates to 1 µmol/L of ascorbate plasma. A constant plateau in ascorbic acid concentration (60–80 µmol/L) is reached at 150 mg of consumed vitamin C [34]. Given the non-linear link between vitamin C consumption and absorption, the converted FFQ blood concentrations were added to the scatterplot for comparison, but were not included in the analysis. Additionally, ascorbate CSF concentrations were not included in the analysis due to a non-linear relationship with plasma vitamin C.

Additionally, qualitative analyses were conducted on the studies that utilized a range of other cognitive assessments and direct plasma vitamin C measures. These studies were reported qualitatively due to a large diversity in cognitive assessments and statistical reporting of results (odds ratios, confidence intervals, etc.). The overall trend of results and quality of these trials was taken into account for the qualitative analysis.

## 3. Results

**Figure 1:**
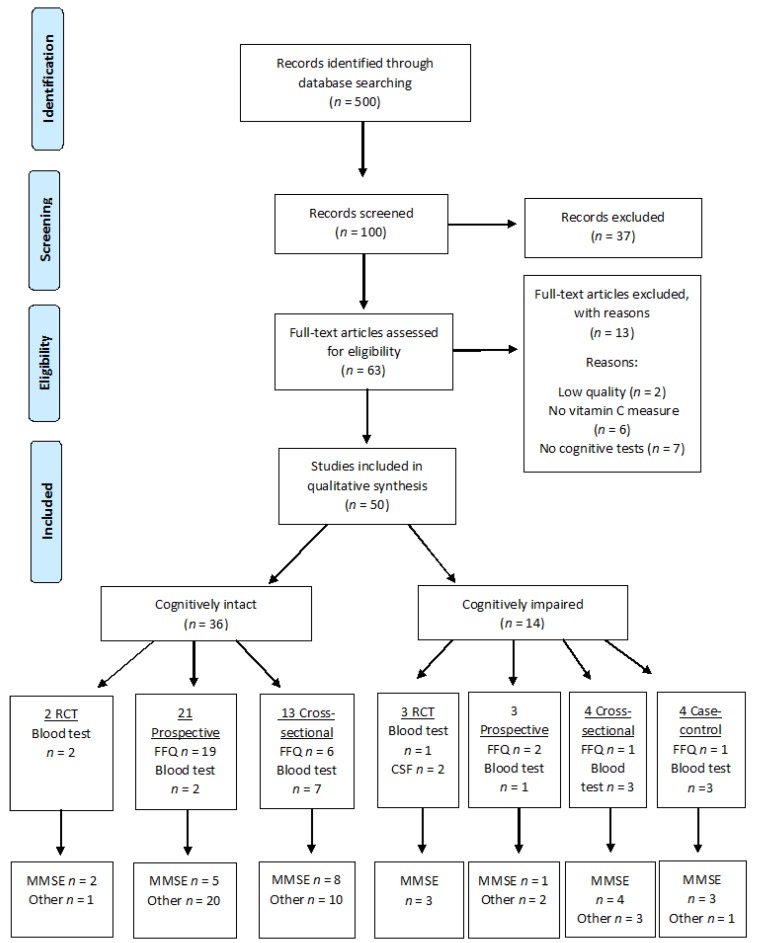
Flow chart of steps in systematic review.

The search captured exactly 500 articles, of which 50 studies were included in the systematic review (Figure 1). Of these, 14 studies involved cognitively impaired participants, e.g., dementia including Alzheimer’s disease and 36 studies were conducted on cognitively intact participants. The cognitively impaired subgroup included 3 RCTS [37,38,39], 4 prospective [26,40,41], 4 cross-sectional [42,43,44,45] and 4 case-control [46,47,48,49] studies (Table 1). The cognitively intact subgroup included 2 RCTS [50,51], 21 prospective [52,53,54,55,56,57,58,59,60,61,62,63,64,65,66,67,68,69,70,71,72], 13 cross-sectional [73,74,75,76,77,78,79,80,81,82,83,84,85], and no case-control studies (Table 2). Table 3 summarises the trials that were excluded from the review, and the reason for their exclusion.

In the cognitively impaired samples, eight out of 14 studies used blood tests to measure vitamin C [26,39,42,43,44,46,47,48], two used CSF [37,38] and four used FFQs alone [40,41,45,49]. A series of cognitive tests were conducted in these studies. Eleven studies [26,37,38,39,42,43,44,47,48,49] used the MMSE and six [37,40,41,42,45,49] used alternate forms of cognitive assessment. In the cognitively intact samples, 11 out of 36 used blood tests to measure vitamin C status [50,51,52,53,76,78,79,80,81,82,83], and 25 studies conducted FFQs [54,55,56,57,58,59,60,61,62,63,64,65,66,67,68,69,70,71,72,73,74,75,77,84,85]. A series of cognitive tests were conducted in these studies. Fifteen studies [50,51,54,55,56,57,58,73,74,77,78,81,82,84,85] used the MMSE and 31 studies [50,52,53,54,55,56,58,59,60,61,62,63,64,65,66,67,69,70,71,72,73,74,75,76,78,79,80,81,82,83,84,98] used other forms of cognitive assessment.

**Figure 2:**
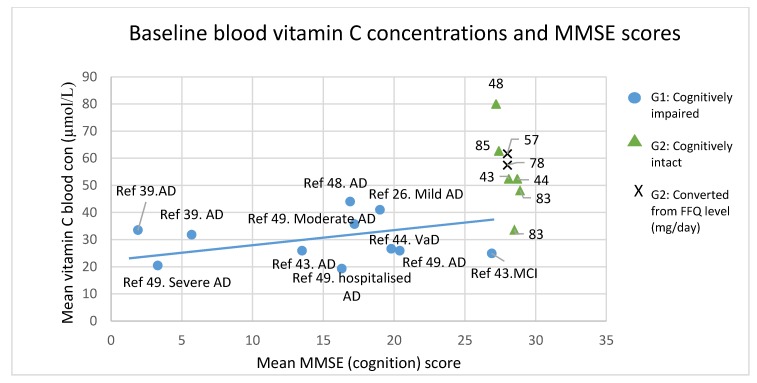
Scatterplot of baseline mean MMSE scores against blood vitamin C concentrations. Blue circles represent cognitively impaired groups of participants, and green triangles and crosses represent cognitively intact groups (triangles: direct plasma vit C measure, cross: converted from FFQ). No correlation analyses were conducted on the cognitively intact data points. The blue line represents the correlation slope amongst the studies of cognitively impaired groups of participants (r_s_ (11) = 0.009, *p* = 0.98). Key: Ref = study reference, * Not included in the analysis, AD = Alzheimer’s disease, CSF = Cerebral Spinal Fluid, FFQ = Food Frequency Questionnaire; MCI = mild cognitive impairment, mg/day = milligram per day, VaD = Vascular dementia, Con = concentration, MMSE = Mini Mental State Examination.

Mean MMSE scores and measured or derived blood vitamin C concentrations are plotted in Figure 2 and presented in Table 4 and Table 5. In the cognitively impaired group, these means were extracted from seven studies (sample sizes ranged from 12–88 participants, with a total of 391 participants). Independent samples *t*-tests revealed that mean vitamin C concentrations in the cognitively intact subgroup were significantly higher than in the cognitively impaired (*t* (15) = 4.5, *p* < 0.01) and mean MMSE scores were also significantly higher in this subgroup (*t* (10.3) = 5.7, *p* < 0.01).

In the cognitively impaired subgroup, there was a wide distribution of both MMSE scores (mean score range = 1.9–26.9) and vitamin C concentrations (19–44 µmol/L) (Figure 2). Mean vitamin C concentration (Mean score ± standard deviation (SD) = 29.91 ± 8 µmol/L) corresponded with a borderline vitamin C depletion (<28 µmol/L) [33]. Mean MMSE scores (Mean score = 14.63 ± 7.8) corresponded to a severe cognitive impairment (scores >17) [99].

In the cognitively intact subgroup, mean vitamin C and MMSE scores were extracted from 5 studies (sample sizes ranged 18–260 participants, with a total of 496 participants). In this group, mean vitamin C concentrations (Mean score ± SD = 54.9 ± 16) µmol/L) were widely spread (33.7–80 µmol/L) but mean MMSE scores (Mean score = 28.1 ± 0.7) were not (27.2–28.9). The lack of variance in MMSE scores precluded correlational analysis in this subgroup.

In the cognitively impaired subgroup the scatterplot (Figure 2/Table 4) and a Pearson r^2^ value of 0.0016 revealed low variance and a spread in means around the fitted regression line. The Spearman’s correlation also revealed no significant correlation between MMSE scores and vitamin C concentrations (r_s_ (11) = 0.009, *p* = 0.98).

A number of studies [44,50,78,81] (Table 5) did not report numerical mean vitamin C concentrations or MMSE scores (0–30) but instead placed the means into categories (e.g., MMSE score of over/under 27, Vitamin C concentrations into deficient/adequate ranges). The results from these studies followed our observed trend where participants whose vitamin C concentrations were categorized into adequate ranges produced higher mean MMSE scores and those who were categorized into scoring under 27 on the MMSE had lower mean vitamin C concentrations.

Additional studies using cognitively intact groups of participants (Table 2) assessed cognition using a number of different cognitive measures and plasma vitamin C. Examples of these cognitive measures included the digit span backwards/forwards, the East Boston memory test, Wechsler memory test, clock drawing, delayed word recall, etc. (Table 2). A majority of these studies [50,52,78,79,81] revealed an association between vitamin C blood concentrations and cognitive performance on various cognitive tasks. Some of the cognitive domains included short-term memory, information processing, abstract thinking and working memory. A number of studies [80,82,83] did fail to demonstrate a link between vitamin C and cognition. However, the quality assessment revealed lower ratings for these studies than for those demonstrating a link. Additionally, one study [42] using cognitively impaired groups of participants (Table 1) assessed cognition with alternative assessments to the MMSE and demonstrated superior performance in those with higher vitamin C concentrations.

The predicted blood vitamin C concentrations generated from FFQs in the cognitively intact participants when plotted (Figure 2), were relatively similar to the blood concentrations generated by studies primarily using blood tests. These converted values were not used in correlation analyses.

## 4. Discussion

This review evaluated 50 studies exploring the link between vitamin C and cognitive function. Extrapolated mean vitamin C concentrations and MMSE scores from a number of these studies indicated that the cognitively intact groups of participants had higher mean vitamin C concentrations and MMSE scores than the cognitively impaired groups. However, there was no significant correlation between mean vitamin C concentrations and mean MMSE scores in the cognitively impaired studies (*n* = 7, *n* = 391 participants). In contrast, correlation analysis between blood vitamin C concentrations and MMSE scores in the cognitively intact studies was not feasible due to the low variance in MMSE scores, demonstrating the unsuitability of the MMSE in the cognitively healthy participants. Quantitative assessment of those studies in the cognitively intact groups revealed a potential association between plasma vitamin C concentrations and cognition. Our findings are consistent with a number of studies [42,48,95] that showed a significantly lower vitamin C blood concentrations between cognitively impaired compared to healthy individuals.

This may be explained by a reduction in dietary intake amongst the elderly in general [100], and those living alone or in aged care/hospital facilities [101] who are often unable to prepare their own meals, may have chewing problems, and may make poor food choices such as not including fruits and vegetables in their diet.

Subjects with AD may be nutrient deficient, particularly in the later phase of the disease. However, case-control studies have also demonstrated lower plasma vitamin C concentrations in the early AD stages in well-nourished subjects [48].

A more recent, second hypothesis for the depleted blood vitamin C concentrations in the cognitively impaired is the increased oxidation of vitamin C in response to elevated free radical production in the brain. Vitamin C has been reported to be the first barrier to free radicals produced in biological fluids [102]. In the cognitively impaired, studies have demonstrated an increased sensitivity to free radicals in the cerebral cortex [103]. The mechanisms of free radical production hypothesized for AD include: activated microglia surrounding senile plaques [104], neuronal mitochondrial dysfunction [105], intraneuronal amyloid accumulation [106] and presence of redox active metals [107]. Thirdly, disturbances in iron metabolism found in the vicinity of the senile plaques [108], could catalyse the production of free radicals. Noradrenergic and serotoninergic deficiencies have also been reported in AD [109], requiring the utilisation of vitamin C to restore these deficiencies.

The lack of linearity in vitamin C concentrations and MMSE scores in the cognitively impaired group could be explained by the non-linear relationship between plasma vitamin C and ascorbate CSF absorption. Due to a homeostatic mechanism [26], the amount of ascorbate CSF and vitamin C reaching the brain could show little variability at varying plasma concentrations, even with deficient plasma concentrations (<28 µmol/L). This could result in similar cognitive scores at varying plasma vitamin C concentrations.

### 4.1. Limitations

The results from the current review do need to be interpreted cautiously due to a number of limitations: 

While blood samples are a more reliable measure of vitamin C status than FFQ-based Vitamin C determination, a number of further methodical issues may exist. Many factors can contribute to the instability of ascorbic acid in biological samples due to the oxidation of vitamin C in plasma is accelerated by heat, light, and elevated pH (acidity). These issues arise as a result of a lack of full appreciation of the redox chemistry and biology of ascorbic acid [110]. A number of handling techniques should be incorporated in order to ensure quality measures.

A majority of studies included in this review failed to thoroughly explain blood sample handling and biochemical analysis. Ideal handling conditions of samples intended for ascorbate analysis include immediate coverage from light, immediate plasma isolation, rapid acidification, and freezing below −20 °C to avoid misinterpretations compounded by the use of poorly preserved samples [110]. In order for plasma to be transported, it needs to be covered from light and transported on dry ice (−70 °C) before thawing and analysis.

Underestimation of vitamin C concentrations could occur if samples were not handled properly. Frequent freeze-thaw cycles or exposure to any metals (such as iron in the haemolysis of red blood cells) could both lead to rapid degradation of vitamin C in the sample [111]. It has been shown that there is a significant loss of ascorbate plasma in EDTA tubes [112], with lithium heparin tubes being ideal.

Several limitations can arise from the use of FFQs in determining nutrient level [32]. Plasma vitamin C concentrations are dependent on recent dietary intake, due to the vitamin’s water soluble properties and excretion, therefore blood plasma measures would be most reflective of foods consumed recently (1–2 weeks). Incorporating food questionnaires relating to most recent food consumption, would be most indicative of blood concentrations. Given the overreliance on FFQs in the reviewed studies, especially in those incorporating prospective designs, instead of blood samples interpretation of findings is limited. A direct comparison between FFQ and blood samples could validate the effective of the questionnaire. A recent meta-analysis demonstrated that FFQ and food diaries have a moderate relationship with plasma vitamin C, with multiple factors affecting this relationship [32].

While converted FFQ-based vitamin C levels were of a similar range to blood concentrations, this conversion needs to be interpreted with caution. The conversion ratio of 1.95 mg to 1 µmol/L in plasma was based on a study that used 8 healthy participants [34]. However, this ratio may not be applicable for all individuals as individual factors could affect vitamin C absorption and distribution (i.e., oxidative stress, infection, etc.).

Plasma vitamin C differs according to polymorphisms of sodium dependent active transporters (SVCT2 and SVCT1) despite equivalent vitamin C intake indicating that SVCT1 and 2 genotype may determine the strength of the association between vitamin C intake and circulating vitamin C concentrations [113]. Some people may require greater than the recommended daily allowance to maintain optimal vitamin C concentrations. These differences could render food diary information even less accurate as perceived intake may not be equivalent to absorption [111].

In addition, dietary assessment has reliability and validity issues in relation to even mild cognitive deficits, which are frequent in older populations [114]. These include recall errors but even when food types and amounts are recalled correctly, differences in storage and cooking can decrease the vitamin C level in the food [115]. It is close to impossible to determine the concentrations retained in foods following manipulations such as cooking [116]. Furthermore, high levels of vitamin C gained from dietary sources will often be accompanied by higher levels of a number of other beneficial compounds (vitamins, phytochemicals) also found from the same sources [111].

Moreover, the reviewed randomised controlled studies have failed to assess the effects of a vitamin C intervention on its own, by using multivitamins. A large portion of the included studies have made efforts to statistically control for potential confounders. Although our review did demonstrate lower plasma vitamin C concentrations in the cognitively impaired, other studies using impaired samples have shown depletions in a number of other vitamin and minerals including: vitamin B12 [117], vitamin E [118], vitamin D [119], vitamin K [120], folate [117], and elevated homocysteine [117]. Additionally, it is important to note that when antioxidant function is involved, vitamins can work synergistically with other vitamins, e.g., vitamin C recycles α-tocopherol radical (vitamin E) [111]. The consumption and supplementation of these vitamins should be considered as potential confounders and should be monitored, especially in cognitive impaired participants.

Moreover, it can be speculated that a consistently high Vitamin C status acts in a preventive manner, while vitamin C supplementation per se is not a treatment for clinical AD [48]. Thus, infrequent supplement users may not achieve the same benefits as individuals with consistent intake of adequate vitamin C. Controlling for vitamin C supplementation use, or taking it into account, is crucial.

Intake at the time of measurement may not reflect lifetime dietary habits and given data that suggest that amyloid plaque burden begins to form well before middle age [121], intakes during younger adulthood may be equally as important as supplements taken by older adults, perhaps contributing to a biological buffer against disease pathogenesis. Measuring and controlling for a history of consumption and supplementation is crucial, especially in longer prospective studies where the development of neurodegeneration is being investigated.

In addition to the limitations on vitamin C levels, there were limitations regarding the type of cognitive measures. A number of long term prospective studies incorporated cognitive tests suitable for screening and assessing the incidence of Alzheimer’s, such as the MMSE. Given the simplicity of such tests, and the scales used to measure performance, it becomes difficult to establish cognitive changes unless the cognitive decline is extremely severe. These MMSE scales have been effective in measuring cognition in those clinically diagnosed with a neurodegenerative condition [96,48], and were useful in the cognitively impaired subgroup in this review.

The sensitivity of the MMSE to detect differences in cognitively intact samples has been questioned [122,123]. This can lead to a lack of variance in MMSE scores. In our review, the mean MMSE score ranged 27.2–28.9 in this group (<24 = mild cognitive impairment). In this review, a number of studies conducted on the cognitively intact group did use a range of other, more suitable cognitive tests, including the digit span forwards/backwards, delayed word recall, letter digit substitution test, etc., with mixed results. A number of these studies [55,67,70,74,83] failed to demonstrate a link between vitamin C status and cognition whereas a number of studies [50,61,76,79,81] demonstrated the effects of vitamin C on a number of cognitive domains such as free recall, short-term memory, abstract thinking, visuospatial performance and recognition. However, comparison of different cognitive tests was beyond the scope of this review.

A further limitation to be considered is the often self-selection of healthier, more cognitively-able population in population studies. As a consequence of high baseline performance in cognitively intact participants, ceiling effects with narrow ranges in results can occur [124]. This effectively minimizes several confounding factors, but narrows the chance of detecting cognitive effects.

In cognitively intact samples, cognitive tests sensitive to age-associated cognitive decline should be employed to maximize the observation of any potential effects. Programs such as The Cambridge Neuropsychological Test Automated Battery [125] and The National Institute of Health (NIH) Toolbox [126] are available that tap into a wide range of cognitive domains sensitive to change from mid adulthood such as fluid intelligence would be ideal for establishing its association with nutrition or intervention [127]. In the present review, one study [79] using cognitively intact participants incorporated a computerized test battery assessing a number of cognitive domains. This study demonstrated a significant link between vitamin C status and free recall, recognition and vocabulary.

### 4.2. Future Directions

Future studies should incorporate a number of recommendations. Firstly, the most reliable and practical measure of vitamin C is the measurement of biological blood samples. Moreover, the incorporation of FFQs would allow a measure of possible confounding variables (vitamin B12, vitamin E, etc.). Age-sensitive cognitive tests assessing response time and accuracy should be administered [127], particularly in the case of cognitively intact individuals. A number of potential confounding factors such as supplementation, and the long term intake of other vitamins and minerals associated with cognition need to be take into account.

## 5. Conclusions

In summary, studies included in this systematic review demonstrated higher mean vitamin C concentrations in the cognitively intact groups of participants compared to the impaired groups. No correlation was found between vitamin C concentrations and MMSE scores in the cognitively impaired groups of participants. Analysis of the studies that used a variety of cognitive assessments was beyond the scope of this review, however, qualitative assessment in the cognitively intact groups revealed a potential association between plasma vitamin C concentrations and cognition. Due to a number of limitations, further research, assessing plasma vitamin C concentrations, taking confounding factors such as vitamin B12 and vitamin E into account, and the use of more sensitive cognitive assessment methodology for cognitively intact participants are needed to provide more insights into the relationship between vitamin C and cognition.

## Figures and Tables

**Table 1:** Characteristics and outcomes of studies using cognitively impaired samples.

Paper	Study Design	*N*	Age (years)	Condition	Quality Rating	Cognitive Measure	Vitamin C Measure	Outcome
Arlt, 2012 [37]	RCT	23	60–80	AD	6	MMSE, Word fluency, Immediate/delayed verbal recall, Trail-making task	CSF	1000 mg/day of vit C and E (400 mg/day) increased CSF concentrations after 1 year, but decreased MMSE score and no effect on other measures
Galasko, 2012 [47]	RCT	78	50–85	AD	4.5	MMSE	CSF	Decline in MMSE score occurred in E/C/ALA group. (500 mg/day vit C, vit E, alpha lipoic acid) did not influence CSF biomarkers related to amyloid
Burns, 1989 [39]	RCT	81	≥65	Senile Dementia, Community dementia	4.5	MMSE	Blood tests	200 mg Vit C, vits B1, B2, B3 No correlation between vit C intake and cognitive impairment
Bowman, 2009 [26]	Pros	32	71	AD	5	MMSE	CSF, plasma ascorbate	Neither Plasma nor CSF AA predictive of AD across 1 year
Zandi, 2004 [40]	Pros	4740 (4540 healthy)	≥65	AD	3.5	3MS, Dementia Questionnaire (DQ)	Supplement, Interview	vit E (>400 IU) and C (500 mg) supplements reduced the AD prevalence and incidence. Supplements alone had no protective affect across 2 years
Deijen, 2003 [41]	Pros	90	>65	Psychiatry nursing home	4.5	Dutch geriatric nursing scale, Zorg Index geriatrie (ZIG)	Food record	Higher vitamin intakes were associated with a worse daily functioning across 6 months
Rinaldi, 2003 [42]	Cross	141	>70	MCI, AD	3	Clinical dementia rating scale, MMSE, clock drawing test, Babcock story recall, auditory verbal learning test, Corsi block tapping test, Token test, category naming test, Oral word association test, visual search test, digit forward and backward test, Raven’s progressive colored matrices	Plasma ascorbate	Lower vit C concentrations in patients with AD and MCI. MCI sig lower then controls
Polidori, 2004 [43]	Cross	141	≥65	AD, VaD	2	MMSE	Plasma ascorbate	Plasma AA lower in AD and VD
Richardson, 2002 [44]	Cross	37	65–97	In-patient ward	2	MMSE	Plasma ascorbate	75% with dementia had low concentrations of vitamin C
Lu, 2016 [45]	Cross	2892 (768 MCI)	58	MCI	2.5	Montreal cognitive assessment	FFQ	Carotenoids, vit C, and vitamin B6 exhibited the highest protective factor loadings
Charlton, 2004 [46]	CC	93	≥65	Dementia	4	MMSE	Plasma Ascorbate/FFQ	Plasma AA lower in dementia, not explained by diet
Glaso, 2004 [47]	CC	38	75–85	AD	4	MMSE	Serum ascorbate/CSF	Both plasma vitamin C and CSF lower in AD. CSF: plasma AA ratio higher in AD
Riviere,1999 [48]	CC	69	>75	Severe AD, Moderate AD, Hospitalised AD	3.5	MMSE	Plasma ascorbate, FFQ	Nutritional intake lower in Severe AD, plasma vit C lower in more severe AD, not explained by vit C intake
Masaki, 2000 [49]	CC	3735 men	71–93	Dementia	3	Hasegawa scale, MMSE	Self-report supplementation	After controlling for factors such as age, education, stroke, there was an association with cognitive performance

Key: MCI = Mild cognitive impairment, AD = Alzheimer’s, VaD = vascular dementia RCT = Randomized control trial, Pros = prospective, Cross = cross-sectional, CC = case-control, Vit = vitamin, FFQ = food frequency questionnaire, CSF = cerebrospinal fluid, MMSE = Mini mental state examination, 3MS = Modified Mini Mental State Examination, ALA = alpha lipoic acid.

**Table 2:** Characteristics and outcomes of studies using cognitively intact samples.

Paper	Study Design	*N*	Age (years)	Quality Assessment	Cognitive Measure	Vitamin C Measure	Outcome
Chandra, 2001 [50]	RCT	86	≥65	5.5	Wechsler memory test, Halstead-Reitan categories test, Buschke consistent long-term retrieval, digit span forward, salthouse listening span test, long-term memory recall, MMSE	Plasma spectrophotometry	80 mg of vitamin C in a multivitamin improved cognitive performance, not Long-term memory across 1 year
Dror, 1996 [51]	RCT	21	>80	3.5	MMSE	Plasma Assay	No changes in MMSE scores following 42-day supplementation with 45mg/day of vitamin C with other vitamins (Vit D, E B12, B6)
Gale, 1996 [52]	Pros	921	≥65	2.5	Hodkinson mental test (Dementia assessment)	Dietary intake/Ascorbate plasma	Cognitive function was poorest in those with the lowest vitamin C over 1 year
La Rue, 1997 [53]	Pros	137	66–90	5	Abstract performance, visuospatial performance, memory assessment	Plasma Ascorbate, Nutritional status	Visuospatial performance was higher with higher ascorbate concentrations after 6 years
Paleologos, 1998 [54]	Pros	117	69–91	4	MMSE, Reid brief neuropsychological Screen, the animals test of category fluency, the F, A, S test of verbal fluency	Semi-quantitative food frequency	After adjusting for age, sex, smoking, education, energy, vit C supplement linked to less severe cognitive decline, not verbal/category fluency across 4 years
Devore, 2002 [55]	Pros	16,010	>70 Women	5	MMSE, Telephone interview for cognitive status (TICS). East Boston memory test (immediate/delayed) category fluency, Delayed TICS, Digit span backwards	Semi-quantitative food frequency	Dietary vitamin C intake not associated with cognitive decline. Supplemental vit C associated with worse decline over 6 years
Engelhart, 2002 [56]	Pros	5395	>55	3.5	DSM-III-R criteria, MMSE	Semi-quantitative food frequency (SFFQ)	Higher dietary vit C intake associated with less AD after a mean of 6.5 years, controlling for supplements
Kalmijn, 1997 [57]	Pros	342 Men	69–89	3	MMSE	Dietary history FFQ	Higher vit C intake not correlated with cognitive decline or impairment after 3 years
Laurin, 2003 [58]	Pros	2549 Men	45–68	4	Hasegawa dementia screening instrument, MMSE, 3MS	24-h dietary recall	Vit C was not associated with the risk of dementia or its subtypes across an 8-year period
Basambombo, 2016 [59]	Pros	5269	≥65	2.5	Diagnostic and Statistical Manual of Mental Disorders (DSM-III-R)	Self-reported supplementation	The use of vitamin C supplements associated with a reduced risk of cognitive decline during 3, 5 year intervals
Nooyens, 2015 [60]	Pros	2613	43–70	5	15 Words Learning Test, the Stroop Test, Word Fluency test, Letter Digit Substitution Test	178-item semi-quantitative FFQ	No associations between intakes of vit C and cognitive decline across 5 years
Peneau, 2011 [61]	Pros	2533	45–60	4.5	RI-48 cued recall, semantic, and phonemic fluency tests, trail-making and forward and backward digit span tests	24-h dietary record	vit C–rich FVs (P-trend = 0.03), vitamin C (P-trend = 0.005) positively associated with verbal memory across 13 years
Fotuhi, 2008 [62]	Pros	3376	≥65	2.5	3MS	Self-report	Combined vit C, E, and anti-inflammatory resulted in a lower decline on the 3MS across 8 years. Vit C alone had no affect
Gray, 2008 [63]	Pros	2969	≥65	3.5	Cognitive abilities screening instrument	Self-report	No association between vitamin C and AD incidence, or vit C and E together after 2.8–8.7 years
Wengreen, 2007 [64]	Pros	3831	≥65	3.5	3MS	Food frequency	Higher quartiles of vit C intake had a greater 3MS score and lower vit C intake had a greater rate of decline during 7 years
Fillenbaum, 2005 [65]	Pros	616	65–105	3.5	Short portable mental status questionnaire	In home interview	Vitamin C did not reduce AD or dementia incidence over either 3 or 14-year interval
Maxwell, 2005 [66]	Pros	894	≥65	3.5	3MS	Self-report	Subjects reporting supplementation of vit C were less likely to have cognitive decline or to be diagnosed with VCI after 5 years
Grodstein, 2003 [67]	Pros	14,968	70–79 women	4.5	Telephone Interview of Cognitive Status, Delayed recall of 10 word lists, Immediate and delayed recall of paragraph, Verbal fluency, Digit span backwards	Supplementation questionnaire	Vit C and E had higher mean global scores than non-supplemented. Vit C alone did not affect global score after 5 years
Luchsinger, 2003 [68]	Pros	980	≥65	4.5	Neuropsychological test battery	Semi quantitative food frequency	Neither dietary, supplemental nor total intake of vit C across 4 years was linked to AD Incidence
Morris, 2002 [69]	Pros	815	>65	3	Consortium Established for Research on AD	FFQ	Intake of vitamin C was not significantly associated with risk of AD across 3.9 years
Peacock, 2000 [70]	Pros	12,187	48–67	4.5	Delayed word recall test, Wechsler adult intelligence scale, Revised digit symbol subtest, word fluency test	Food frequency questionnaire	No consistent association between dietary and supplemental vit C and cognition across 8 years
Morris, 1998 [71]	Pros	633	≥65	3.5	Criteria for clinical diagnosis	Supplementation questionnaire	None of the vitamin C users were diagnosed after a mean of 4.3 years
Mendelsohm, 1996 [72]	Pros	1059	≥65	2.5	Neuropsychological battery (15 items)	297 vitamin C self-report supplementation	After adjustment for age, race, income, education, vit C supplementation did not relate to cognitive scores during 2 years
Berti, 2015 [73]	Cross	52 Women	54–66	1.5	Clinical dementia rating, Global deterioration score, MMSE	Harvard/Willet FFQ	Antioxidant consumption positively associated with METglc (*p* < 0.001)
Beydoun, 2015 [74]	Cross	1274	30–60	2	MMSE, CLVT-list A, CVLT-DFR, digit span forward/backwards, Benten visual retention test, Animal fluency test, Brief test of attention, trail making test, Clock drawing test, card rotations, identical pictures	Two 24-h recalls	Vitamin C not associated with cognition on either cognitive task, MMSE error count (*p* = 0.17)
Chaudhari, 2015 [75]	Cross	582	40–96	2	Repeatable battery for the assessment of neurological status, The executive interview	Ascorbate supplementation (self-report)	Vit C led to better immediate memory (*p* = 0.04), visuospatial skills (*p* = 0.002), language (*p* = 0.01), global cognition (*p* = 0.006)
Goodwin, 1983 [76]	Cross	260	>60	2	Halstead-Reitan Categories, (Non-verbal abstract thinking), Wechsler Memory Test	Dietary intake/Ascorbate plasma	Performance worse on both tasks in those with low vit C (5–10% lowest levels)
Jama, 1996 [77]	Cross	5182	55–95	2.5	MMSE	Semi-quantitative food frequency questionnaire	No association between cognitive function and intake of vitamin C intake (<70mg/day (odd ratio) = 1.14, 130–160 mg/day (od) = 1.21
Lindemann, 2000 [78]	Cross	195	≥65	3	MMSE, WAIS-R Digits Forward, Fuld Object Memory Evaluation, Clock drawing, Two Color Trail Making Tests	Serum ascorbate	Lower vit C not associated with cognition. There was a trend. Low vit C linked with a history of depression
Perrig, 1997 [79]	Cross	442	≥65	3	Computerised cognitive test (assessed working, implicit and explicit memory), WAIS-R vocabulary test	Plasma Ascorbate	Free recall, recognition, and vocabulary (not priming or working memory) correlated with ascorbic acid concentrations (semantic memory *p* = 0.034, vocabulary test *p* ≤ 0.021)
Schmidt, 1998 [80]	Cross	1769	50–75	2	Mattis Dementia Rating Scale	Plasma (chromatograph)	No association between cognitive scores and plasma concentrations (odds ratio = 1, *p* = 0.87)
Sato, 2006 [81]	Cross	544	≥65	2.5	Digit symbol substitution task (DSST), MMSE	Ascorbate plasma, Block’s FFQ	Highest fifth of plasma ascorbate associated with better DSST, marginally with MMSE
Whalley, 2003 [82]	Cross	176	77	2.5	MMSE, Raven’s Progressive Matrices	Ascorbate plasma, FFQ (MONICA)	No difference between those taking vitamin C supplements and controls, after controlling for childhood IQ, education, socioeconomic status and cardiovascular health
Perkins, 1998 [83]	Cross	4809	>60	2	Delayed word recall, Delayed story recall	Serum ascorbate	After adjusting for socioeconomic factors and other trace elements, vitamin C concentrations were not associated with poor memory performance
Ortega, 1997 [84]	Cross	260	65–90	1.5	MMSE, Pfeiffer’s mental status questionnaire	Food frequency for 7 days	Higher cognition correlated with great vitamin C intake across 7 days
Requejo, 2003 [85]	Cross	168	65–90	0.5	MMSE	Food record	Those with a greater intake of vitamin C were more likely to display adequate cognitive ability

Key: MCI = Mild cognitive impairment, AD = Alzheimer’s, VaD = vascular dementia RCT = Randomized control trial, Pros = prospective, Cross = cross-sectional, CC = case-control, Vit = vitamin, FFQ = food frequency questionnaire, CSF = cerebrospinal fluid, MMSE = Mini mental state examination, 3MS = Modified Mini Mental State Examination.

**Table 3:** List of studies with reasons for exclusion.

Study	Study Design	Reason for Exclusion
Kennedy (2011) [86]	RCT	Mood/fatigue primary measures, vitamin C status not assessed
Smith (1999) [87]	RCT	Self-reported cognitive failures (subjective cognitive assessment)
Kumar (2008) [88]	RCT	Vitamin C status not assessed
Yaffe (2004) [89]	RCT	Cognition not assessed at baseline, vitamin C status not assessed
Kang (2009) [90]	RCT	Cognition not assessed at baseline, only 3.5 years after intervention
Chui (2008) [91]	RCT	Vitamin C status not assessed, no placebo/blinding
Day (1988) [92]	RCT	Vitamin C status not assessed, assessed only confusion
Paraskevas (1997) [93]/Quinn (2004) [27]/Woo (1989) [94]/Polidori (2002) [95]/Foy (1998) [96]	CS	No cognitive tests administered
Talley [97]	Pre-test post-test	Simple orientation/consciousness assessment

Legend: RCT = Randomised control trial, CS = case-control.

**Table 4:** Cognitively impaired participants (Mean blood vitamin C/MMSE scores).

Paper	Study Design	*N*	Mean Vitamin C Level in μmol/L (SD)	Mean MMSE Score (SD)
Burns (1989) [39]	RCT	81	Intervention baseline-33.5 (28) Placebo baseline-31.8 (31) Placebo final-25 (28) ^#^	1.9 (3.3) 5.7 (9.1) 5.7 (10.6) ^#^
Bowman (2009) [26]	Pros	32	41 (30)	19 (5)
Rinaldi (2003) [42]	CS	25 63	MCI-24.9 (2.4) AD-25.9 (8.9)	26.9 (2) 13.5 (6.5)
Polidori (2004) [43]	CS	63 23	AD-25.9 (8.9) Vascular AD-26.6 (11.3)	20.4 (3) 19.8 (3)
Glaso (2004) [47]	CC	20	AD-44 (25)	16.9
Rivierie (1999) [48]	CC	24 9 20	Moderate AD-35.7 Hospitalized AD-19.3 Severe AD-20.4	17.2 (4.9) 16.3 (6.1) 3.3 (3.1)

Legend: SD, standard deviation; RCT = randomised controlled trial, Pros = prospective, CS = cross-sectional, CC = case-control, # not a baseline value therefore not included in analysis, blue circles representing cognitively impaired blood values.

**Table 5:** Cognitively intact participants (Mean blood vitamin C/MMSE scores).

Paper	Study Design	*N*	Vitamin C Level in μmol/L (SD)	MMSE Score (SD)
Engelhart (2002) [56] *	Pros	5395	61.7 (27)	28
Jama (1996) [77] *	CS	5182	57.5	28
Ortega (1997) [84]	CS	260	62.7 (33.5)	27.4 (4.8)
Whalley (2003) [82]	CS	79 31	Non-supplement user-33.7 (26.2) Supplement user-48.2 (25.7)	28.5 (1.4) 28.9 (1.4)
Glaso (2004) [47]	CC	18	Control group-80 (28)	27.2
Polidori (2004) [43]	CS	55	Control group-52.4 (16.4)	28.7 (1)
Rinaldi (2003) [42]	CS	53	Control group-52.4 (16.5)	28.1 (1.4)
Chandra (2001) [50] ^#^	RCT	86	Adequate Deficient	28 (6.3) 17 (4)
Lindemann (2003) [78] ^#^	CC	195	>57 <57	27.2 (2.4) 26.4 (2.9)
Sato (2006) [81] ^#^	CC	544	Median = 74.9 (interquartile range = 57.8–90.7) Median = 78.9 (interquartile range = 64.1–99.2)	<27 >27
Richardson (2002) [44] ^#^	CC	37	<11 11–40 40–100	23 (12.3) 25 (6.0) 27 (5.1)

Legend: RCT = randomised controlled trial, Pros = prospective, CS = cross-sectional, CC = case-control, * converted FFQ to blood vitamin C (μmol/L) represented by crosses on Figure 2 (not included in analysis), green circles representing cognitively intact blood values (Figure 2), ^#^ Not included in analysis.
